# Evaluating research investment and impact at a regional Australian Hospital and Health Service: a programme theory and conceptual framework

**DOI:** 10.1186/s12961-020-0542-y

**Published:** 2020-03-06

**Authors:** Alexandra Edelman, Amy Brown, Tilley Pain, Sarah Larkins, Gillian Harvey

**Affiliations:** 1 James Cook University, Townsville, Queensland Australia; 2 Townsville Hospital and Health Service, Townsville, Queensland Australia; 3 University of Adelaide, Adelaide, Australia

**Keywords:** Research impact, Programme theory, Hospital and Health Service, regional, Queensland, Australia

## Abstract

**Background:**

Health systems in Australia and worldwide are increasingly expected to conduct research and quality improvement activities in addition to delivering clinical care and training health professionals. This study aims to inform a research impact evaluation at a regional Australian Hospital and Health Service by developing a programme theory showing how research investment is expected to have impact.

**Methods:**

This qualitative study, representing the first phase of a larger mixed methods research impact evaluation at the Townsville Hospital and Health Service (THHS), adopts a realist-informed design involving the development of a programme theory. Data were obtained between February and May 2019 from strategic documentation and interviews with six current and former health service executives and senior employees. Inductive themes were integrated into a conceptual framework to visually represent the programme theory.

**Results:**

Research at THHS has developed organically as the service has matured into a regional tertiary referral service serving a diverse rural and remote population across northern Queensland. Throughout this journey, individual THHS leaders often adopted a research development mantle despite disincentives arising from a performance-driven reporting and activity-based funding service context. Impact expectations from research investment at THHS were identified in the categories of enhanced research activity and capacity among clinicians, and improved clinical practice, health workforce capability and stability, and patient and population health. Seven contextual factors were identified as potential enablers or obstacles to these impact expectations and ambitions.

**Conclusions:**

By identifying both relevant impact types and key contextual factors, this study offers programme theory to inform a planned research impact evaluation at THHS. The conceptual framework may be useful in other regionally based health service settings. More broadly, there are opportunities for future research to test and refine hybrid versions of linear and realist research impact evaluation models that combine resource-intensive, theory-driven approaches with policy practicality.

## Introduction

Health systems in Australia and worldwide are increasingly expected to conduct research and quality improvement activities in addition to delivering clinical care and training health professionals [1, 2]. In Australia, achieving the vision of “*better health through research*” [2, 3] requires investment in different types of biomedical, services and systems research that involve engaging practicing clinicians in the research process to maximise health returns for patients and populations [3]. A growing body of evidence links research activity in clinical settings with better healthcare and outcomes [4–7]. The Townsville Hospital and Health Service (THHS), a statutory body overseeing healthcare delivery across a vast area of regional and remote northern Queensland, has sought to build this research function over two decades by investing in research infrastructure and capability.

Evaluations of research impact can offer insights into how resources are being used and can inform strategic planning [8]. Evaluations can also help to align research efforts with priority population or service needs [2]. However, defining the ‘impacts’ of health research is notoriously challenging – definitions wrestle with notions of different types of impact (whether health-related, social or economic), types of beneficiaries (whether individuals, organisations or communities), or proximity to the research endeavour such as the degree to which an impact can be attributed to a specific research activity [8]. Reflecting the range of possible interpretations of ‘impact’, research impact evaluations and assessments employ a range of different frameworks, most of which are variations of logic models that attempt to link research inputs and outputs to downstream impacts [8, 9]. Buxton and Hanney’s well-known Payback framework, for example, enables the identification of impacts across multiple categories from knowledge production through to more distal health and economic returns [10, 11]. Theory-driven approaches can also be employed to identify what combinations of mechanisms and contexts produce different types of research-related impacts in particular settings [12]. Many impact frameworks also incorporate narratives to illustrate impact in a qualitative way and as perceived by the end-user [8].

Over the recent decade, research impact assessments have started to be incorporated into funding and research quality assessment schema based on perceived and documented impact or research translation. This has occurred in multiple countries, including Australia, the United Kingdom, Canada and the United States [13–16]. However, despite substantial policy interest in measuring research impact, the evaluation and recording of the impacts of research on policy and practice in Australia is often narrowly focused on easy-to-measure outputs, such as counts of peer-reviewed publications, and is rarely undertaken systematically [17]. Such output-focused metrics on their own are insufficient to determine the value of research investment because they overlook potential real-world impacts and benefits [18]. Ideally, comprehensive research impact evaluations should take account of different evaluation aims and contexts, potentially involving the development of tailored impact evaluation approaches or frameworks [17]. This study aims to inform a research impact evaluation suited to a regional Australian health service setting by developing a programme theory showing how research investment was expected to have impact within THHS.

## Methods

### Study setting

**Fig. 1:**
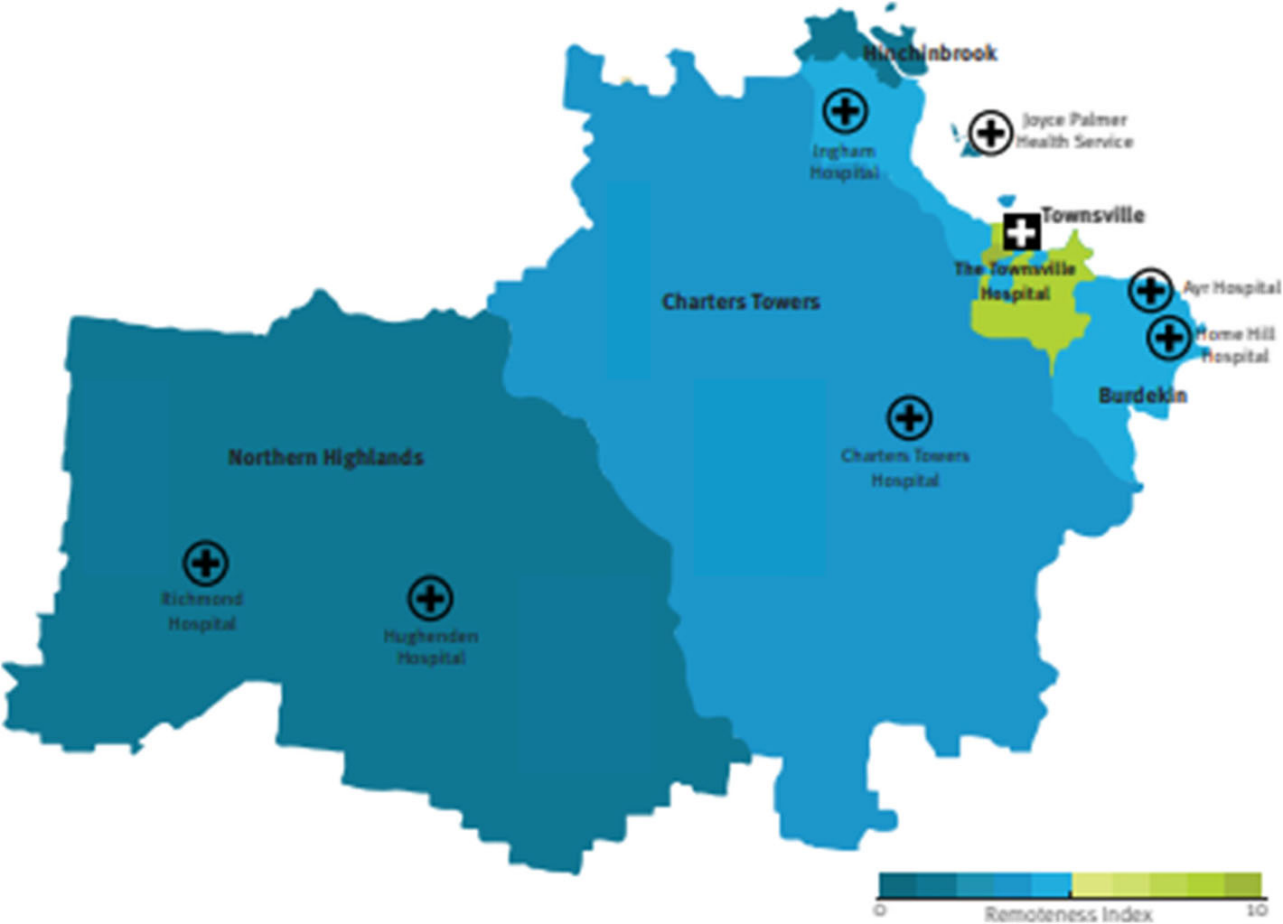
Geographic boundary of the Townsville Hospital and Health Service (THHS) by remoteness area (source: THHS Operational Plan 2018–2028)

The THHS serves a diverse population of around 250,000 people across an area of approximately 149,500 km^2^ in northern Queensland, Australia (Fig. 1). The THHS comprises 21 facilities across its catchment, including 19 hospitals and community health campuses and 2 residential aged care facilities. Approximately three-quarters of the population reside within the regional city of Townsville and approximately 8% identify as Aboriginal and/or Torres Strait Islanders. The Townsville Hospital, the THHS tertiary referral hospital, has 590 beds and treats patients from across northern Queensland. The hospital supports a referral catchment of almost 700,000 people from some of Australia’s most remote communities – located as far as the Cape York Peninsula and the Torres Strait Islands in the north, and Mount Isa and the Gulf of Carpentaria in the west. Large areas of THHS are classified as relatively disadvantaged. The region has higher rates of chronic diseases, such as diabetes, chronic obstructive pulmonary disease, coronary heart disease and stroke, than the rest of Queensland and Australia, with diabetes, cardiovascular, mental health and chronic kidney disease responsible for higher hospitalisation rates among the Aboriginal and Torres Strait Islander population.

### Design

This qualitative study, representing the first phase of a larger research impact evaluation at THHS, adopts a realist-informed design involving the development of a programme theory. This approach draws from realist evaluation principles, which consider not only project outcomes but also what works, for whom, and in what circumstances – how and why [19]. Programme theories represent an understanding about how certain resources can influence patterns of behaviour or action [20]. Explicitly documenting assumptions about how a programme is expected to work offers a starting point for future investigation of what is being implemented, how the programme is being implemented, and the “*mechanisms of impact*” – how the programme brings about change in particular contexts [21]. This study identifies key contextual factors influencing an intended impact pathway; the evaluation project to follow this initial study aims to examine context–mechanism–outcome configurations in greater depth.

### Data collection

Data were obtained between February and May 2019 from documentation and interviews. Documentation relating to research strategy and investment at THHS were collected and interviews were conducted with current and former senior THHS employees who were involved in the research investment journey at THHS.

#### Documentation

Categories of potentially relevant documentation were identified by the research team based on existing knowledge of governance and planning structures within THHS, with additional documents then sought iteratively. Access to relevant documentation not publicly available was requested from the THHS Chief Executive. A total of 18 research-specific and generic strategy documents that focused on research development at the whole-of-organisation level (i.e. not at discipline level) were collected from the earliest dates available to currently operational documents. These documents were two research strategies (2014–18; 2018–22), one research operational plan (2018–19), two research annual reports (2015–16; 2017–18), four strategic plans (2007–12; 2012–16; 2014–18; 2018–22), six annual reports (2012–13; 2013–14; 2014–15; 2015–16; 2016–17; 2017–19), one external consultancy report on research governance (2013), and two Queensland Health–THHS Service Agreements (2013/14–2015/16; 2016/17–2018/19).

#### Interviews

To gain insights into the strategies and expectations underpinning research investment at THHS, interviewees were purposively selected with reference to their degree of involvement in research investment at senior levels, period of employment at THHS and discipline (professional) background. Seven potential interviewees were identified and were contacted by email by the THHS-based researchers. As one individual was unavailable due to being on extended leave, semi-structured, in-depth interviews were undertaken one-on-one with six current and former THHS executives and senior employees. These interviews averaged 52 min duration and were conducted during April and May 2019. All interviewees had been involved in research investment at THHS at various periods in a research development journey. Three interviewees offered insights into research development at THHS from a whole-of-organisation perspective on issues relating to organisational strategy, board expectations and research-enabling infrastructure (e.g. research ethics and governance processes), with a further three offering insights from distinctly nursing, allied health and medicine perspectives. Four of the interviewees offered insights into current developments and future plans, two of whom also reflected on historical developments over two decades. A further two interviewees reflected on their experiences and observations around the 2008–2014 period. The history of involvement of some members of the research team in different research-related activities of THHS over several years meant that the team had existing knowledge of the key roles and personnel over time, which helped in selecting and contacting interviewees. However, care was taken to ensure that no potential interviewees were excluded based on prior knowledge of their possible views. All interviewees were provided with a participant information sheet and signed an informed consent form.

Five interviews were conducted in person and one was conducted by phone. Interviews were undertaken concurrently by two members of the research team (AE and AB) who took turns acting as lead interviewer and observer. The role of the observer was to ask follow-up questions and to take written notes of any implicit meanings or interpretations that may have been missed by the lead interviewer. Interviewees were asked about their current and/or former roles at THHS, their involvement in research development, and their perspectives on the strategic priorities of research investment, key milestones, outcomes and challenges (see interview schedule at Additional File 1). Interviews were digitally recorded and transcribed in full by two researchers (AE and AB), with copies of transcripts then sent to interviewees for checking.

### Data analysis

Documents and interview transcripts were coded inductively by two members of the research team (AE and AB) using NVivo software (QSR 12.3.0). Multiple members of the research team read a sample of the transcripts to discuss an emerging coding framework, with two researchers (AE and AB) independently coding a transcript and then discussing the coding process and handwritten notes from interviews to refine the codes and discuss emerging themes. Codes from documents and interviews were then compared and documents re-coded for consistency.

To inform the development of the programme theory, the research team first identified key milestones and features of a research investment and development journey at THHS and reported these in narrative form. A list of aspirational indicators of impact was then developed and grouped into impact types. Inductive themes were then developed representing the causal assumptions between the types. Following this, contextual conditions relevant to the linkages were identified in each theme. Interview data was used initially to inform this analysis, with documentation used to supplement emerging explanations. The themes were then integrated into a conceptual framework to visually represent the programme theory. At this point, two researchers (AE and AB) met with senior health service managers who had not yet been involved in the study to verify the framework as a starting point for the planned research impact evaluation. This involved seeking their perspectives on whether there were any other types of impact or contextual factors that the research team had not yet considered but which should be built into the framework. These discussions emphasised certain aspects of the study findings, which were subsequently considered in the presentation of the framework. Finally, the framework was compared with seven impact frameworks and approaches (Table 1) to identify similarities and differences. These comparator frameworks represent some of the most established or important emerging research impact approaches as identified in a narrative review on research impact by Greenhalgh et al. [8]. Three additional frameworks were identified by the research team through searching of reference lists and web searches and were included for their relevance to the context of this study.

**Table 1:** Comparator research impact frameworks and approaches

Framework	Key features
The Payback Framework [10]^a^	This framework is one of the most widely used in research impact assessments and informs many of the newer approaches. The framework consists of a logic model of the seven stages of research from conceptualisation to impact, and five categories of impacts, called ‘paybacks’: knowledge production, research targeting and capacity-building, informing policy and product development, health and health sector benefits, and broader economic benefits. The framework incorporates various feedback loops connecting the stages.
Health Services Research Impact Framework [17]	This framework was developed in an Australian primary healthcare context and identifies four ‘broad areas of impact’: research-related impact (advancing knowledge), policy impact (informing decision-making), service impact (improving health and health systems), and societal impact (creating broad social and economic benefit). Against each broad area, the framework lists specific areas of impact, reach into different audiences, and whether impact involves ‘producer push’ dissemination or ‘producer pull’ uptake.
Canadian Academy of Health Sciences Impact Framework [9]^a^	This framework adapts the Payback Framework (above) into a ‘systems approach’ to capture impacts in five main categories: advancing knowledge, research capacity-building, informing decision-making, health impacts, and broad economic and social impacts. Each main category consists of subcategories containing lists of possible indicators, which can be used to track impacts within the four ‘pillars’ of health research: basic biomedical, applied clinical, health services and systems, and population health. The framework also allows tracking of impact at individual, institutional, provincial, national or international levels.
Impact Assessment Tool [18]	This framework groups different types of impacts into ‘four levels of impact’ with subcategories: scholarly outputs (publications and citations, research funding, capacity-building, journal impact factor), translational outputs (plain language summaries and media engagement, formal knowledge exchange processes, lobbying government ministers or departments, intervention packaged for implementation), policy or practice impacts (changes to practice, changes to services, policy change, commercialisation), and long-term population outcomes (behaviour change and health outcomes, social outcomes, economic outcomes).
Alberta Innovates-Health Solutions Research to Impact Framework [22]	This framework adopts the same impact categories as the Canadian Academy of Health Sciences framework above but additionally highlights ‘inputs’ (involving stakeholder engagement and evidence to inform planning and investment strategies). The framework also incorporates performance management concepts in the form of ‘balanced scorecard’ categories (financial, enablers, internal processes and stakeholder) and logic model categories (inputs, activities, outputs, reach, short term outcomes, medium-term outcomes and long-term outcomes). These categories are mapped against the subcategories of organisational performance, research and innovation outcomes, informing decision-making, health and wellbeing, and broader economic impacts.
Realist evaluation [8]^a^	Rather than representing or offering an impact framework, realist evaluation is presented in the narrative review by Greenhalgh et al. [8] as an approach that holds future promise in research impact assessment. Realist evaluation addresses the question: what works, for whom, and in what circumstances? [19] Realist evaluation models highlight the role of context in influencing outcomes.
SPIRIT Action Framework [23]^a^	The purpose of this framework is to “*guide action including the identification of where, how and what should be done to help agencies improve the use of research in their work*”. While the framework uses a logic model structure as in many other impact frameworks, its focus is more on the receiving organisation’s need for research rather than on the research itself. The framework commences with catalysts for research use, and follows through to capacity, research engagement actions and outcome. ‘Outcome’ is divided into outcomes associated with research use, research-informed health policies and policy documents, and finally with better health systems and health outcomes. The framework also proposes hypotheses to enable the examination of whether the upstream components of the logic model result in changes further downstream.

^a^These frameworks and approaches are described in a narrative review by Greenhalgh et al. [8]

## Results

Results are first reported as a narrative description of the research development journey at THHS. A programme theory is then described showing causal assumptions between the investments and various types of impact, which includes key contextual factors influencing impact outcomes.

### Research development at the Townsville Hospital and Health Service (THHS)

The history of research investment at THHS is interwoven with its broader development journey and tropical, rural and remote location and population focus. Key elements of the journey included initial co-location of the Townsville Hospital with the Australian Institute of Tropical Medicine – one of the earliest research institutes in Australia – and then broader development of the health service from a regional general hospital through to its current role as the main tertiary referral service for the whole of northern Queensland (THHS Strategic Plan 2018–2022). The Australian Institute of Tropical Medicine closed in Townsville in 1930, with a hiatus prior to the opening of the Anton Breinl Centre for Tropical Medicine in the same building in 1987 and moving to its current home on the James Cook University (JCU) campus health precinct in 2004. Research functions within the Townsville Hospital in the 1990s were nascent and evolved organically over the subsequent decades:

*“The* [research] *journey is largely an organic one – you can’t go from zero to 100 – we had to go from being a large regional hospital to a tertiary health service, and that took time. That took going from one surgeon deep in a specialty to two, or one physician deep to two or three. And then, from there, growing a sub-specialty base*.” (Int 5)

The THHS’s transition to a tertiary service paralleled the development of the health faculty of JCU, for which the Townsville Hospital is a major clinical teaching site. The relocation of the hospital from its previous inner-city location to adjacent to the JCU campus in 2001 was a key milestone in the development of the health service, enabling growth of the hospital’s clinical as well as research and teaching functions and capabilities. Co-location with JCU’s health and medical faculty solidified the role of the Townsville Hospital as the major teaching hospital of JCU and supported collaboration between the two organisations in research (THHS Annual Report, 2012–13). The establishment in 2017 of a North Queensland Academic Health Hub Alliance built on this history of collaboration and co-location by signalling an intent between the two organisations to create “*a world class teaching and learning centre with a focus on research and innovation*”, aimed at positioning THHS as “*the largest clinical teaching and research entity in northern Australia*” (2017–18 Annual Report). With JCU, the THHS is one of the founding partners of the Tropical Australian Academic Health Centre, a collaboration which brings the THHS and JCU together with the four other Hospital and Health Services (HHSs) in northern Queensland and the Northern Queensland Primary Health Network around a translational research agenda. As one of the three stated pillars of the THHS mandate since 2014, research is an important THHS strategic priority underpinned by the idea that by improving research engagement of clinical staff, the THHS can ultimately improve clinical care and patient and population health across the northern Queensland region (THHS Research Strategy 2018–2022).

From the early 2000s, research investments largely occurred independently in the different professions, with separate development trajectories in medicine, nursing and allied health. In medicine, two professorial-level roles in surgery were established and filled in 2002 as part of a joint hospital-JCU research workforce development framework. This represented an approach to research workforce development that involved appointing research leaders as conjoint appointments with JCU:

“*To augment the research component we worked with the university – they actually had a template of where they saw clinical academic roles growing over the subsequent years … it was basically a core set that we wanted to develop into* [ … ] *We were working off that framework for probably the next two-to-three years, looking at where the next appointment should be.*” (Int 6)

Further senior research appointments in medicine included a Clinical Dean and a Director of Clinical Research, who have worked with JCU to lead research development in medicine since 2010. Concurrently, the 2012 Medical Officers (Queensland Health) Certified Agreement, which included a requirement that 10% of medical staff time be spent on “*clinical support*” activities, was seen to have been influential in encouraging medical clinicians to engage in research (Int 1).

In nursing, a key enabling appointment of a Nursing Manager for Research was made in 2002 to encourage the engagement of nurses and midwives in designing and undertaking research. A professorial appointment in nursing was also made as a joint JCU-THHS appointment in 2007. In contrast to medicine, research development in nursing mostly involved up-skilling of existing clinical staff rather than new senior-level appointments. A key development, emblematic of this approach, was a requirement established in 2001 that all clinical nurse consultants in the hospital needed to complete an “*action research project*” annually if they were to retain their positions and develop their careers. This initiative was a product of the vision of the then Director of Nursing:

“*I think what* [the nursing director] *really wanted them* [the clinical nurse consultants] *to do was to look at their routine practices and look at the myths – do we just do this because we’ve always done it this way? Why have we always done it this way? So it was about making – trying to get nurses to question their practice. Everyday things. Not to do huge research – that was never the intention*.” (Int 4)

Although this requirement involved some challenges and had been phased out, it contributed to establishing an “*academic approach*” to clinical practice within nursing (Int 6). In both nursing and allied health, research workforce development was encouraged by a one-off provision in 2008 of “*workforce development*” funding, which was used within these disciplines to give clinicians time to engage in research activity. A key appointment in allied health was an Allied Health Research Fellow in 2010, which was established with Queensland Government funding as part of a state-wide programme. This was followed by the first professorial allied health appointment in 2011, but this position was discontinued in 2014, when the incumbent left and there was limited funding available to retain the role (Int 1).

Among the professions, nursing was the first to establish a research event for staff, which commenced in 2002 to coincide with International Nurses’ Day. A similar research showcase event was established for allied health in 2009, with medicine-specific events subsequently established in 2011. These events were combined into one whole-of-organisation “*research week*” in 2012, which continues to be held annually to enable clinical staff to present their work and network with colleagues around research opportunities. Now known as the annual THHS Research Showcase, this event is a key strategic action within the THHS Research Strategy (2018–22) and is reported on in the research annual reports:

“*The annual showcase is a unique opportunity to demonstrate health research taking place across the health service and academic campus with an emphasis on collaborative research which harnesses the collective power of both institutions. It provides an excellent opportunity for staff who may have the beginning of an idea for research to meet people who can help turn their dream into a reality*.” (THHS Research Annual Report 2017)

The showcase events also offer skills and methodology workshops, including in publication writing, statistics and health economics. JCU’s cohort-based programme for higher degrees by research students, established in 2011, also opened opportunities for THHS staff to undertake more formal research training in a supportive environment part-time while still working clinically. The development of the JCU MBBS Honours programme in 2005, frequently involving supervision by THHS clinicians, similarly contributed to the availability of formal research training options and linkages for medical professionals.

Direct funding for research activity at THHS commenced in the early 2000s from a trust fund administered by the health service, with disbursements initially led at service group level. The first formal research funding round for medicine was initiated in 2004, which was later followed by annual competitive funding rounds open to all THHS staff. In addition to these internal funding sources, clinicians engaged in research at the THHS access funding from the Queensland Government (including the New Technology Funding Evaluation Program and Health Practitioner Research Scheme) and other bodies such as the Emergency Medicine Foundation, the Royal Australian and New Zealand College of Obstetricians and Gynaecologists, and private insurance companies. Over time, several THHS researchers have developed sufficient track records to access funding from highly competitive sources such as the National Health and Medical Research Council, with the first such grant awarded to a THHS vascular surgeon in 2003.

Establishment of the Townsville Research, Education, Support and Administration unit in 2015 was aimed at strengthening research ethics and governance processes within the THHS and added organisational capability in research data management and reporting. The first published THHS research strategy that adopted a whole-of-organisation focus was produced in 2014 and the first formal research annual report was published in 2016. The 2019–2022 THHS Research Strategic Plan articulates a vision for THHS:

“*…to be the leading hospital research centre in northern Australia, translating novel research into innovative, high-quality patient care.*” (2019-2022 THHS Research Strategic Plan)

The Research Operational Plan 2018–19 outlines an approach to make ongoing research investments more discerning and strategic through the development of research themes and more targeted funding, overseen by a Research Development Committee chaired by the Director of Clinical Research. A dedicated space for research within the Townsville Hospital, called the Townsville Institute for Research and Innovation, was also launched in early 2019.

### Research impacts, causal assumptions and key contextual factors

**Fig. 2:**
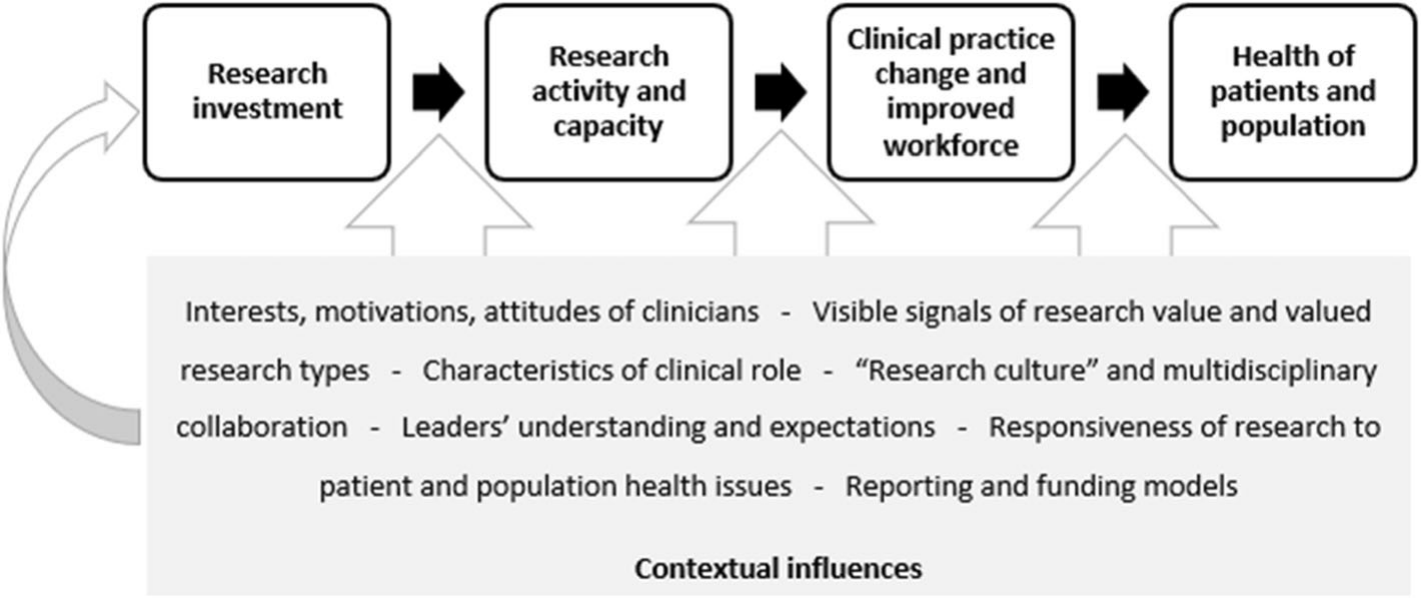
Conceptual framework showing causal assumptions between research impact types and key contextual influences

Research investments at the THHS, described above, have taken multiple forms and have aimed to create an infrastructure that both promotes and enables research activity within the health service. By creating a set of circumstances to enable various types of impact to occur, these investments collectively represent a purposeful strategy intended to shape the actions of clinicians and health service managers within the health service. Three causal assumptions between the investments and expected impacts were identified (Fig. 2). In the first instance, the research investments were expected to increase research activity and capacity among THHS clinicians. In turn, these were expected to lead to better patient care and a more capable and stable health workforce, which was then expected to lead to patient and population health improvements. These assumptions form the basis of a programme theory describing how returns from research investment at the THHS were expected to occur. The elements of the programme theory, forming the coding tree for this analysis, are discussed below and include seven contextual factors that appeared to act as the main enablers or barriers to the intended theory, producing both intended and unintended outcomes. There was a high degree of overlap and interdependency between the contextual factors, as shown in Fig. 2. The sections below describe the research impact types followed by the contextual factors that appeared to most closely affect these impacts. The research impact evaluation structure arising from this analysis is provided in Additional File 2.

### Theme 1: research investment promotes and enables research activity and capacity

THHS documentation and comments by interviewees suggested that THHS research investments were expected to have a direct impact on the research activity and capacity of clinicians employed within THHS facilities. Research activity expectations were expressed in the academic indicators of impact in the THHS Research Annual Reports, and included numbers of publications, grants, projects and clinicians involved in research (2015–16; 2017–18). Some disciplines collected data on research capacity impacts, and the THHS Research Annual Reports reported on capacity-building investments such as the number and type of research training programmes, workshops and events delivered across the HHS, which implied an expectation that clinicians’ research skills and capabilities would improve (2015–16; 2017–18). Interviewees also described expected capacity-building impacts from research investments, which included greater opportunities for research-related career progression and increased research skills among clinicians (Int 1; Int 4). One interviewee described the quality of research-enabling infrastructure as a type of capacity-building impact that should be considered in thinking about returns from research investment:

“*We have a well-regarded research governance framework. And we only have that because actually we’ve been at this research thing for a while, we’ve learned along the way, we’ve got bumps and cuts and bruises, you know. So that in itself is a research impact sort of piece.*” (Int 5)

As such, expected capacity-building impacts involved a broad range of indicators, including workforce factors such as clinicians’ research literacy and skills and organisational factors such as quality and extent of available research-enabling infrastructure and career pathways. Several contextual influences appeared to enable or hinder these expected impacts and represented an interplay of intrinsic (individually motivated) factors and organisational features and structures.

#### Contextual factor 1: clinicians’ interests, motivations and attitudes

Clinicians’ interests and motivations to engage in research appeared to be a key factor influencing both the quantity and quality of research activity outputs. The experience in the THHS of making research activity mandatory in nursing revealed that the degree to which clinicians were intrinsically motivated to do research was a key factor in determining whether and how they took up research opportunities. While some nurses gained satisfaction from the requirement, others appeared to resent being made to do research and either participated just to “*tick the box*” or did not do the research:

“*Some nurses were performance-managed out of their roles because they didn’t do it* [ … ] *others really engaged with it, got the idea* [that] *research is really good* [ … ] *and others just did rubbish stuff like create a form and said that’s research*.” (Int 4)

Similarly, if research investments did not take account of personal motivations and learning styles, they risked failing to engage clinicians in research. For instance, although the establishment of senior research roles was a type of investment that often enhanced the support available to clinicians to initiate and lead their own studies, individuals in these roles could unintentionally discourage clinicians’ research engagement if clinicians’ preferences or ways of working were not considered (Int 2). Another interviewee similarly reflected that it was essential, although sometimes challenging, for research training and support investments to take account of individuals’ learning needs:

“*You get very experienced doctors who are clinicians who aren’t experienced researchers – and that’s potentially a recipe for disaster because they are experienced clinicians* [who need] *to be nurtured through in a very respectful manner. And it can be quite tricky at times*.” (Int 3)

Responsiveness to individuals’ interests, motivating factors and approaches to learning is therefore likely to be necessary for research investments to have activity and capacity impacts.

#### Contextual factor 2: visible signals of research value and valued research types

The degree of visibility of research investments appeared to influence clinicians’ research motivations by serving as signals that research was valued or not by the organisation. For example, the establishment of senior research roles was seen to have had the effect of making research seem “*credible*” to clinicians and therefore worth being involved in, because the organisation had valued it enough to commit resources (Int 4). Investment in physical research spaces and enabling infrastructure were similarly described as both physical spaces that could be used by researchers as well as tangible signs that the organisation supported research:

“[The space] *is emblematic – the concept was always, make it easy for people to do research as part of their day’s work, to try and give a sense that the organisation applies importance to it*” (Int 6)

Conversely, an absence of such signals, such as of research-related presentations at orientation events, was seen to convey a message to staff that the organisation did not support research, which in turn could create a barrier to research engagement (Int 4).

In some cases, however, visible signals of research investment had the potential to hinder research engagement. For example, organisational promotion of valued research types had the potential to discourage other, less-promoted, types of research. Two interviewees reflected that a focus in the THHS on measuring narrow research impacts, such as publications and grants, inadvertently encouraged and rewarded medical research over allied health and nursing research, which were less represented in these types of outputs (Int 1; Int 4). Further, investments could sometimes be made that signalled support for research without actually enabling real research activity and capacity-building to occur:

“*To me it’s a waste time if departments have a journal club and things like that and they go – oh yeah look what this person did – but then not actually giving the ability or enabling their staff to actually undertake those projects themselves*.” (Int 2)

Although visible signals of research infrastructure, support and activity often encouraged research engagement, they also had the potential to hinder research activity and capacity impacts if they focussed on narrow research types or masked inaction to address more structural barriers.

#### Contextual factor 3: characteristics of an individual’s clinical role

The degree to which clinicians were able to engage in research was influenced by the characteristics of their clinical role, including workload, requirements of their profession and status in the organisation. Despite inclusion of research-related provisions within professional awards and career structures, many challenges were apparent in the “*grow your own*” approach to research workforce development within THHS (involving upskilling existing clinical staff) (Int 6). For example, regardless of a clinicians’ personal interest or motivations, or availability of research workshops and events, if a clinician’s service manager was not supportive of their research involvement they were sometimes faced with the option of undertaking the research work out of hours “*for no overt reward*” (Int 1). While it was generally observed by interviewees that most service group leads were supportive of staff doing research – and were getting more supportive over time – there was still a sense that staff in THHS were battling against a history wherein “*research was the poorer cousin of everything else*” (Int 2).

Although several clinicians at the THHS had developed research careers, one interviewee observed that this tended to be ad hoc and varied both within and between professions (Int 2). Interviewees described generally limited research-related career opportunities for staff who had engaged in research training:

“*At the moment we have a lot of people going off to do PhDs – so they do their PhD or they do their research and then they come back and there’s no opportunity to grow … they just go back to their clinical position and quite often they’re back in that thing where there’s no protected time to do research*” (Int 2)

Without defined career pathways, some clinicians who were involved in research did not have research functions formally described in their position statements, and these clinicians sometimes struggled to balance their research activities with their substantive clinical roles. Finding the time to do research alongside clinical duties was described as a particular challenge:

“*Although we say in theory that research is up there, that doesn’t sometimes cross into clinical practice because of the clinical workload of a lot of our people who want to do research –* [they] *just don’t have the capacity to be able to do it*.” (Int 1)

Individual clinicians’ capacity to make time for research was described by some interviewees as differing by profession, with nurses described by one interviewee as less able to find discretionary time for research than other clinical staff (Int 4). Allied health professionals were also described by another interviewee as facing an uphill battle to do research because the value of allied health research was less understood by senior staff in the organisation (Int 1). This interviewee described a need for allied health research to be constantly defended, whereas medically led research was more taken-for-granted as worthwhile. As well as creating challenges for individuals, limited formalisation of research as part of clinicians’ roles also presented an organisation-level risk to growing research activity and capacity because recruitment needed to be to a purely clinical role regardless of the research involvement of an incumbent (Int 6).

### Theme 2: research activity changes clinical practice and improves clinical workforce capability and stability

Beyond research activity and capacity-building, research investments at the THHS were expected to impact on clinical practice behaviour and clinical workforce capability and stability. Indeed, the idea that research outputs could be translated into evidence-based patient care was a key element of THHS’s research vision (THHS Research Strategy 2018–2022). Interviewees similarly emphasised the role of research at the THHS in improving clinical care, which one interviewee suggested might manifest in “*models of service delivery informed by research*” (Int 5). Interviewees also expected increased research capacity and activity to enhance the capability and stability of the THHS clinical workforce. Research was described by one interviewee as a useful aid for recruitment of senior clinicians to the health service:

“*Despite huge numbers of new-ish grads, it’s difficult to recruit experienced clinicians in many of the professions, particularly outside of metropolitan areas. So if you’re competing to get an experienced senior clinician in whichever of our professions, the ability to contribute in research* [ … ] *you can engage them in that way*.” (Int 1)

A research-capable workforce was also described as a marker of a more clinically capable workforce, with one interviewee suggesting that the clinical capabilities at the THHS would not be as strong if research was not a part of the health service:

“*You look at the workforce that we’ve got and their research output* [ … ] *if we didn’t have that cadre of researchers and clinicians, would we be at the same level of clinical output? And the answer’s no*.” (Int 5)

Two contextual factors appeared important in enabling or hindering these intended impacts – the type of culture at the THHS and the expectations of executive-level staff about what research was able to deliver in the health service.

#### Contextual factor 4: ‘research culture’ and multidisciplinary collaboration

THHS’s two published research strategies both emphasised the importance of establishing a “*research culture*” that not only encouraged clinicians’ interest and involvement in research but that also promoted the integration of research activity with clinical practice (2014–2018; 2018–2022). The 2014–2018 THHS Research Strategy lists “*foster a vibrant research culture*” as a key objective, which involved opening access to research investment to all staff and encouraging diverse types of research engagement across the health disciplines. The desired culture was described in the Strategy as “*vibrant*”, “*strong*” and “*imaginative*” as well as enabling of collaborative effort:

“*It is important to maintain and build a cohesive, supportive, collegial research culture – a culture that values its members and being part of the culture is both fulfilling and rewarding*.” (THHS Research Strategy 2014–2018)

The 2018–22 Research Strategy similarly describes the need for a culture that is “*values-based*” and that embraces not only research but also the notion of “*innovation*” (2018), and the 2018–2019 Research Operational Plan describes the desired culture as one of “*continuous improvement*” (2018). These stated attributes of a desired research culture at the THHS suggest that a certain way of thinking and working is important to enable research activity to be relevant and applied to clinical practice. One interviewee explained that the sort of culture needed was one in which clinicians could draw on research skills to question their practice and then identify and test alternative approaches:

“*The intent of the* [research] *workforce is to contribute in a broader way than I am treating this patient now.* [Research] *is an enabler – a sort of vehicle that allows people to translate that question they had on the floor, into some answer in a structured way*.” (Int 1)

Enabling clinicians to challenge existing practice by accessing or generating new knowledge was thought of as a key step in linking research activity and capacity with improvements in clinical care, because it promoted “*evidence-based practice*” (Int 3). Multidisciplinary teamwork appeared to be an essential ingredient: one interviewee suggested that a questioning mindset among clinicians was best developed and enacted in multidisciplinary teams, by harnessing “*the collective brains trust*” for the benefit of patients (Int 1). This interviewee suggested that research collaboration between clinicians across the disciplines enabled interactions that were sometimes usefully provocative (Int 1). The goal of encouraging “*collaborative, multidisciplinary research*” was also emphasised in the 2018–2022 Research Strategy, and the important role of teamwork similarly highlighted in the 2014–2018 Research Strategy:

“*The best research is done in teams, especially strategically constituted teams where different members bring complementary expertise and where more senior members mentor less experienced researchers*.” (THHS Research Strategy 2014–2018)

Some of the research investments such as research showcase events and workshops appeared to directly enable this kind of productive multidisciplinary interaction. However, the largely separate research development pathways and cultures of the different disciplines suggested that a multidisciplinary, team-based research culture was not yet fully realised in the THHS.

#### Contextual factor 5: leaders’ understanding of research

The degree of understanding among THHS executive and board-level leaders about what research is and what it can deliver for the health service appeared to influence their expectations about the types and timeframes of impact. Low levels of understanding appeared to result in unrealistic expectations among some leaders, which potentially hindered impact opportunities:

“[The individual] *said: ‘show me how this investment translates into that outcome there’. And it’s much more complicated than that. It’s not a direct, linear* [path] *… it doesn’t work that way. But that was extraordinarily difficult to get across*.” (Int 6)

Another interviewee suggested that the biggest risk came from leaders who had little understanding of research but who thought they did. These individuals could sometimes expect research to deliver something that research, by its scientific nature, could not:

“*They* [the leaders] *want research to show that what they’re doing works.* [But] *we’re not doing it to prove it works* [ … ] *I say, ‘we’re not pre-empting any results – you might be disappointed that the findings* [show] *no improvement, no change’*.” (Int 4)

Low levels of understanding among leaders about what research could do within the health service was also seen to contribute to a disproportionate focus on ‘hard’ metrics, like publications and grants, which did not fully reflect research impact possibilities that were hard to measure directly or were less tangible (Int 1). A leadership educated in the possibilities and realities of research and impact therefore appears to be an important enabler of clinical practice impacts.

### Theme 3: ultimate impacts are on patient and population health

Ultimately, the impacts of the research investments were expected to be realised in the health of patients and the region’s population. The articulated purpose statement of THHS emphasised the interconnected nature of clinical care, research and education, with the three pillars combining to ultimately improve health (THHS Strategic Plan 2018–22). Two key contextual conditions were identified as hindering or enabling this aim – the degree to which the research efforts responded to patient and population health concerns, and the features of the THHS regulative environment that either incentivised or discouraged research as part of a definition of “*health system performance*”.

#### Contextual factor 6: responsiveness of research effort to patient and population health concerns

The extent to which clinical impacts from research in the health service translated to broader population-level impacts appeared to depend on whether and how research was prioritised to patient health issues as well as to broader population health and health system needs. Although a process had recently commenced within the THHS of developing research priorities and targeted funding rounds, research at the THHS had historically been investigator-led rather than aligned with priority research themes. One interviewee reflected that there was a need for prioritisation of research investment and activity to areas that mapped to patient and population health needs:

“[The research effort] *needs to be targeting the real needs …* [targeted] *to health priorities and particular regional priorities, and what the population of Townsville need researching, and what the people of Townsville need.*” (Int 3)

Another interviewee suggested that a “*strategic umbrella*” was needed over the top of clinical research at the THHS, suggesting a need not only for disease-based prioritisation of research but also for types of research that addressed systems issues that spanned the streams of medicine, nursing and allied health (Int 1). “*Health services*” and “*systems*” research were described as having the potential to improve health by investigating how to improve the functioning of the health system to benefit the community:

“*At a HHS* [Hospital and Health Service] *level it’s about leveraging the* [government] *department to say, well we need to do this together and do it differently, so fund us differently, we’ll deliver different outputs because we need to deliver different outcomes for our communities*.” (Int 5)

Health services and systems research as well as clinical research prioritised to identified clinical needs, were therefore potential enablers of patient and population health impacts from the research effort.

#### Contextual factor 7: reporting and funding models

As a statutory entity with reporting and funding relationships with the Queensland Government, the THHS was responsible for delivering on a set of key performance indicators (KPIs) and received public funding through an activity-based funding model. The extent to which THHS met its KPIs and funding targets was understood by interviewees to be a key factor in determining the health system’s “*performance*”:

“*When people talk about ‘health system performance’ they* [focus on] *the easy things to measure, the operational outputs. Meeting your time targets, your volume targets*.” (Int 6)

However, interviewees themselves thought of “*health system performance*” as being more than what was measured and funded by government, and this broader perspective appeared to be necessary for research to be understood by these individuals as valuable. For instance, while statements about the value of research and expectations about research engagement were included in the Service Agreements with the Queensland Government, none of the KPIs directly related to research (2013/14–2015/16; 2016/17–2018/19). One interviewee stressed that, while many of the expected returns from research were not captured in the KPIs, they were nonetheless very valuable to the health service:

“*The reward* [from research] *is less in our KPIs, so we won’t be made a Level 1-performing Hospital and Health Service based on our research achievements. The reward stuff comes from being seen as a leader, being able to recruit leading clinicians, because you are a leader in health service research. And the recognition of pioneering models of service arrangements*.” (Int 5)

Although research was seen by one interviewee as potentially leading to “*monetary savings*” in the HHS (Int 1), the pressures acting on the THHS to meet its service-based KPIs appeared to disincentivise research investment and research-related activities. One interviewee reflected that the funding of research from recurrent/operational budgets meant that research investment competed with service delivery, positioning it, for example, “*in direct competition with delivery of hip replacements*” (Int 3). This was seen to have put significant pressure on service managers in the THHS to concurrently support research while meeting productivity targets, with clinical services often paramount:

“*A challenge for service managers is balancing clinicians’ time between being in the theatre delivering weighted activity units and being productive, versus then supporting them to have time out of the theatre to do research. We get measured about ten different ways about productivity, access, all these things –* [but] *no one’s going to come and say ‘oh its ok that you’ve got a waiting list because you’re doing health services research’. So, it produces a competing interest at the service management level that needs to be worked through*.” (Int 5)

Despite stated support for health service-driven research from governments, research involved a cost to the THHS and was not formally rewarded as part of its core business. This apparent misalignment between expectations and incentives appeared to be a major risk to THHS’ research aspirations, as both research investment and impacts were heavily reliant on the vision of individual leaders and financial latitude within the THHS to commit the required resources.

## Discussion

This study describes the research development journey at a regional Australian HHS and identifies a programme theory showing types of aspirational research impacts and how these impacts were expected to occur. The conceptual framework and impact evaluation structure developed in the study will inform a planned research impact evaluation at the health service.

Aspirational impacts associated with research investment at the THHS were enhanced research activity and research capacity among clinicians, clinical practice changes, improved health workforce capability and stability, and patient and population health benefits. The elements of the programme theory are presented in a conceptual framework that identifies seven contextual factors acting as enablers or barriers to the functioning of the intended theory, some of which appeared to have the potential to hinder or even derail the health service’s research impact expectations and ambitions. By identifying both impact types and contextual factors, this study and the planned research impact evaluation contribute to an identified need for empirical examination of how “*learning healthcare systems*”, that is health systems that intertwine production and implementation of evidence with routine healthcare, are enacted in practice [24], including how they deliver “*value*” in clinical settings [25].

A research agenda that included both clinically focused and health services and systems research prioritised to patient and population health needs was identified in this study as a potential enabler of the aspirational patient and population health impacts. Further upstream, workplace elements that appeared essential for research activity, capacity and clinical practice impacts included a context that promoted a questioning mindset among clinicians, visibly valued and promoted multidisciplinary research of varying designs, offered tailored learning experiences, and provided time and resources for clinicians’ research involvement and career progression. A recent study on allied health research capacity-building at the THHS similarly identified ‘time’ as the most important barrier to allied health practitioners’ engagement in research [26]. A vast body of literature also documents the benefits of, as well as the challenges involved in, interdisciplinary working to effect real-world impacts, whether in science [27], clinical care [28] or health research [29, 30]. This study encapsulates these various enablers and barriers into a set of key contextual factors that warrant consideration in discussions about research impact in the health sector.

Although presented as distinct forces acting on different parts of a hypothetical impact pathway, the contextual factors were highly interrelated and appeared to have varying influence on different research impact outcomes. For example, clinicians’ interests, motivations and attitudes to learning, identified as key contextual influences on research engagement among clinicians, were shaped by a combination of personal preferences, workplace cultures and relationships, as well as external factors such as industrial awards and broader cultures of the different health disciplines. Further, reflecting the cultural and structural differences between medicine, nursing and allied health, the influence of the contextual factors was also different between the disciplines: what was an enabler to some could be a barrier to others. For example, research-reporting schema that relied heavily on publication and grant counts as indicators of research activity and capacity seemed to act as an enabler for medical research but simultaneously undervalued and potentially disincentivised nursing and allied health research.

Analysis of the research development journey and expectations around impact also revealed multiple, overlapping drivers underpinning and sustaining research investment and impact over time. These drivers were both top-down (e.g. making research mandatory in nursing) and bottom up (e.g. staff taking up research training opportunities). They were also externally driven (e.g. research being built into industrial agreements) and internally driven (e.g. some disciplines following the lead of others). A key internal driver and enabler of research investment and impact appeared to be the presence of individuals in the health service who understood research, held strong research visions and were in positions to influence investment. Throughout the research development journey in the THHS, examples were apparent of individual leaders taking on a research development mantle despite this often not being an expected part of their role and despite the disincentives arising from a performance-driven reporting and activity-based funding context. Such reliance on senior individuals to drive and sustain research investment and impact and appears to be a risky strategy given the high executive-level workforce turnover rates experienced in the THHS region [31].

Like several research impact frameworks used as comparators in this study (Table 1), the programme theory depicts a logic model trajectory from direct academic outputs and capacity-building, through to impacts on clinical practice and health. This linear model appears to be the dominant lens through which research impact is conceptualised among health system managers and policy-makers [32]. Like the Alberta Innovates – Health Solutions Research to Impact Framework [22], the programme theory also identifies research inputs (“*research investment*”) as a component of the model. Unlike the logic model-based frameworks, however, the programme theory reflects the realist-informed approach adopted in the study by incorporating several contextual factors that appeared to influence outcomes at each stage of the impact pathway. Indeed, a recognised benefit of realist evaluation approaches is their ability to “*open the black box*” of knowledge translation to practice in complex real-life interventions [33]. Perhaps reflecting the unique health workforce challenges experienced in regional and rural health service settings, the programme theory also identifies aspirational impacts on health workforce stability and clinical capability, which were not clearly identified as impact types in the comparator frameworks. There are opportunities for future research to test and refine hybrid versions of linear and realist research impact evaluation models that combine nuanced, but resource-intensive, theory-driven approaches with policy practicality.

### Strengths and limitations

This study offers an important contribution to the literature by developing an empirically derived conceptual framework for a research impact evaluation in a regional Australian HHS. In addition, the detailed narrative description of the research development journey at the THHS offers an insider perspective on the way that research functions and capabilities have been nurtured in a regional tertiary health service over two decades, which is likely to offer a useful reference for other health services, particularly those in the early stages of building research functions. The comparison of the study’s findings with other published frameworks in the literature is also likely to be useful for health sector managers involved in funding and evaluating research and research impact. Limitations of the study include the small number of interviewees, which may have inhibited the ability of the study to compare perspectives of different groups. However, interviewees were purposively selected across multiple relevant attributes and interviews were in-depth, offering rich and complex findings. Initial study findings were also discussed with a wider group of THHS managers, which helped in clarifying and presenting the final programme theory and conceptual framework.

## Conclusion

Research at the THHS has developed organically as the health service has matured into a regional tertiary referral service serving a diverse rural and remote population in northern Queensland, Australia. By identifying both relevant impact types and key contextual factors, this study offers a programme theory to inform a planned research impact evaluation at the health service.

## Supplementary information

**Additional file 1.** Interview schedule.

**Additional file 2.** Research impact evaluation structure with indicators.

## Data Availability

Data sharing is not applicable to this article as only qualitative datasets were generated and analysed during the current study. The interview schedule used in the study is available as an Additional File.

## Abbreviations

HHS: Hospital and Health Service
JCU: James Cook University
KPI: key performance indicator
THHS: Townsville Hospital and Health Service
