# Supplementary material for: Evaluating research investment and impact at a regional Australian Hospital and Health Service: a programme theory and conceptual framework
Source: Health Res Policy Syst. 2020 Mar 6;18:30. doi: 10.1186/s12961-020-0542-y (PMC7059332; doi:10.1186/s12961-020-0542-y)
Supplement: Supplementary file 1 — Additional file 1. Interview schedule. [file 12961_2020_542_MOESM1_ESM.docx]

**Additional File 1: Interview Schedule**

| 1. Can you tell us about your current/former roles/engagements at Townsville Hospital and Health Service (THHS) and your involvement in the development of research in the organisation? |
| --- |
| 2. Can you describe the main strategic objectives of the research investment at THHS? |
| 2a. How were these determined? |
| 2b. How is/was progress against these objectives being measured? |
| 2c. Have the goals/objectives of the research investment changed over the past 10 years? |
| 3. What are some of the key milestones in the research development journey at THHS? |
| 3a. What do you think drove these developments? |
| 3b. Were the research strategies based on evidence/case studies/prior experience? |
| 3c. Can you give any examples of research projects or initiatives that are/were underway? |
| 4. Have the outcomes from the research investment strategies been as you expected? |
| 4a. If not, why do you think that is? |
| 4b. Have there been any unexpected or unanticipated outcomes? |
| 5. When you think about health system performance at THHS, what do you think are some of the key indicators? |
| 6. Do you think research has a role to play in helping THHS to improve health system performance? |
| 6a. If so, how? If not, why not? |
| 7. Have you come across any major barriers or challenges? |
| 7a. Where barriers were encountered, what was done to try to overcome them? |
| 8. With the benefit of hindsight, is there anything that you would do differently if you were starting out again? |
| 9. What information do you think would be beneficial to know in an evaluation of research investment at THHS? |
| 10. Is there anything else you want to add? |
