# Supplementary material for: Evaluating research investment and impact at a regional Australian Hospital and Health Service: a programme theory and conceptual framework
Source: Health Res Policy Syst. 2020 Mar 6;18:30. doi: 10.1186/s12961-020-0542-y (PMC7059332; doi:10.1186/s12961-020-0542-y)
Supplement: Supplementary file 2 — Additional file 2. Research impact evaluation structure with indicators. [file 12961_2020_542_MOESM2_ESM.docx]

**Additional File 2: Research Impact Evaluation Structure with Indicators**

| **Theme 1. Investment in research-enabling infrastructure both promotes and enables research activity** | **Contextual factors enabling or hindering impact aspirations** |
| --- | --- |
| Indicators of research investment |  |
| *Capital investment in research spaces and equipment* | Clinicians’ interests, motivations and attitudes  Visible signals of research value and valued research types  Characteristics of an individuals’ clinical role  “Research culture” and multidisciplinary collaboration  Leaders’ understanding of research  Responsiveness of the research effort to patient and population health concerns  Reporting and funding models |
| *Human resources* |  |
| *Funding for research projects* |  |
| *Research workshops and training programs* |  |
| *Events* |  |
| *Research-related strategic plans* |  |
| Indicators of research activity impacts |  |
| *Publications* |  |
| *External grants* |  |
| *Research projects* |  |
| *Quality improvement projects* |  |
| *Inter-institutional collaborations* |  |
| *Prizes and awards* |  |
| Indicators of research capacity impacts |  |
| *Research-related professional development (e.g. involvement in training programs, promotions, mentoring/supervisory roles, journal editorships, invited speakers)* |  |
| *Leveraged research funding* |  |
| **Theme 2. Research activity changes clinical practice and improves the clinical workforce** |  |
| Indicators of clinical practice and policy impacts |  |
| *Decision-making impacts (e.g. use of research in clinical guidelines; research cited in health professional education material)* |  |
| *Clinical practice impacts (e.g. researcher-reported use of findings in health care and other areas; validated evidence of adoption of research findings)* |  |
| *Policy influences (e.g. membership of boards, policy committees, funding decision-making bodies, advisory groups; consultations to policy-makers; requests for research to support policy)* |  |
| Indicators of health workforce impacts |  |
| *Clinical capability and stability (e.g. workforce more clinically capable as a result of research; enhanced recruitment capability)* |  |
| **Theme 3. Ultimate impacts are on patient and population health** |  |
| Improved patient and population health impacts |  |
| *Patient outcomes* |  |
| *Population health outcomes* |  |
